# Antimicrobial Antioxidant Polymer Films with Green Silver Nanoparticles from *Symphyti radix*

**DOI:** 10.3390/polym16030317

**Published:** 2024-01-24

**Authors:** Aiste Balciunaitiene, Viktorija Januskevice, Sandra Saunoriute, Urte Raubyte, Jonas Viskelis, Patrick B. Memvanga, Pranas Viskelis

**Affiliations:** 1 Institute of Horticulture, Lithuanian Research Centre for Agriculture and Forestry, 54333 Baptai, Lithuania; viktorija.januskevice@lammc.lt (V.J.); jonas.viskelis@lammc.lt (J.V.); pranas.viskelis@lammc.lt (P.V.); 2 Research Institute of Natural and Technological Sciences, Vytautas Magnus University, 40444 Kaunas, Lithuania; sandra.saunoriute@vdu.lt; 3 Life Sciences Center, Vilnius University, 10257 Vilnius, Lithuania; urte.raubyte@gmc.stud.vu.lt; 4 Centre de Recherche et d’Innovation Technologique en Environnement et en Sciences de la Santé (CRITESS), Faculty of Pharmaceutical Sciences, University of Kinshasa, Kinshasa B.P. 212, Democratic Republic of the Congo; patrick.memvanga@unikin.ac.cd

**Keywords:** green synthesis, *Symphyti radix*, silver nanoparticles, phytochemical analysis, antibacterial activity, antioxidant activity

## Abstract

Antimicrobial natural polymer film with silver nanoparticles (AgNPs) biosynthesized using aqueous plant root extracts as reducing capping agents and for film formatting show extensive applicability for pathogenic microorganism problems. The formation of AgNPs was confirmed by transmission electron microscopy (TEM) and scanning electron microscopy–energy-dispersive spectroscopy (SEM–EDS) techniques. The antimicrobial activity of biofilm with green AgNPs was analysed by inhibiting the growth of Gram-negative and Gram-positive bacteria culture using the Kirby–Bauer disk diffusion susceptibility test. Total phenolic content and antioxidant activity were slightly higher in aqueous extracts of *Sym. Radix* than in *Sym. Radix*/*AgNPs*. The antimicrobial effect of polymer film/AgNPs against selected test bacteria cultures was substantially more robust than with pure film. Pictures of AgNPs obtained by TEM revealed the presence of spherical-shaped nano-objects with an average size 27.45 nm. SEM–EDS studies confirmed the uniform distribution of metal nanoparticles throughout the biopolymeric matrix. Morphological studies of the surface showed that the obtained surface of the films was even, without holes or other relief irregularities. These apparent *Symphyti radix* polymer film/AgNPs’ biological functions could provide a platform for fighting pathogenic bacteria in the era of multi-drug resistance.

## 1. Introduction

Healthcare-associated infections (HAIs) are an immense global issue and a threat to more than 100 million patients each year [1,2]. These infectious diseases are also called nosocomial diseases and can be induced by bacterial growth and colonization on implants, catheters, and other medical devices used in treatment procedures [1,3,4]. Nosocomial pathogens appear in hospital settings and are transmitted through contact with infected patients, healthcare staff, contaminated water, and food [5,6]. HAIs include infections such as catheter-associated urinary tract infections, ventilator-associated pneumonia, central line-associated infections, infections caused by hospital-acquired *C. difficile* infections, as well as infections occurring at surgery sites [2,5]. HAIs pose a major threat to patients’ health and well-being by prolonging their hospital stay, increasing the possibility of complications and mortality rates. Nosocomial infections may also cause additional financial distress and expense not only to the patient but to the healthcare institution as well. Hence, antibiotics have been and still are invaluable tools in treating and preventing many illnesses and infections, including HAIs. However, decades-long misuse, overuse, and overprescription of antibiotics has resulted in an antibiotic-resistant bacteria crisis [7]. Resistance genes can result from inheritance, mutations, and horizontal gene transfer (HGT), allowing gene transfer even between species [8,9]. Due to natural selection and evolution, antibiotic-resistant pathogens have more of a chance of staying viable and passing down these antibiotic-resistance genes to other bacteria, causing bacterial infections to become less susceptible to treatment and more difficult to control [10]. There are currently around 700,000 antibiotic resistance-related deaths each year, and by 2050, this number may increase to 10 million [11,12]. Hence, with the medical benefits of antibiotics decreasing rapidly, researchers have identified nanomaterials as promising alternative antibacterial agents [13]. Nanomaterials and nanoparticles have emerged as innovative antimicrobial materials for treating or preventing infectious diseases. These materials show strong antimicrobial activity against resistant bacteria due to their high surface area-to-volume ratios, resulting in higher ratios between atoms on the surface and atoms on the inside of materials in comparison with corresponding bulk materials [14,15]. Antibacterial materials used in nanomorphology include nonmetal or metal nanoparticles (NPs) such as silver, copper, gold, bismuth, and metal oxide NPs such as TiO_2_, CaO, Fe_2_O_3_, or Al_2_O_3_ NPs. Most of these nanostructured materials show strong antibacterial effects. Due to their unique optical, electrical, and chemical properties, silver nanostructures are widely used in various industries, but most often in medicine and healthcare. Nanomaterials can be synthesized through many methods, such as electrochemical synthesis [16], sonochemical techniques [17], microwave-assisted green synthesis [18,19], and other physical, chemical, and biological approaches [20]. In today’s context, the world is leaning more towards eco-friendly and sustainable ways of synthesizing nanoparticles, rather than chemical methods that may produce toxic waste or have a negative impact on the environment [21,22]. Hence, biological synthesis has become a more prominent way of synthesizing metal nanoparticles. Biosynthesis can be achieved through the use of microorganisms such as bacteria, fungi, and algae, as well as other green reagents found in plant extracts [20,22,23].

*Symphyti radix* (*Boraginaceae*; *Sym. Radix*), also known as comfrey, is a medicinal plant that has been used in traditional medicine for centuries. Comfrey root can be prepared for medical use as a liquid extract, tincture, or ointment [24,25]. Preparations of comfrey roots are known for their therapeutic analgesic effects and regenerative properties. Comfrey root ointments and extracts can be used externally to lessen pain and inflammation of joints and muscles and as a treatment for strains, bruising, and broken bones [26,27,28]. Tea can be used as well to treat liver conditions and gastritis [29]. The root of comfrey consists of allantoin (0.6–4.7%), mucilage (29%)—consisting of polysaccharide glucose and fructose units—phenolic compounds such as rosmarinic acid (0.2%), chlorogenic acid (0.012%), caffeic acid (0.004%) and derivatives, glyceropeptides, and triterpene saponins [28,30]. However, the main compounds that may be responsible for comfrey’s therapeutic effects are rosmarinic acid and allantoin [30]. Multiple studies have shown that rosmarinic acid has many great healing properties, including anti-inflammatory, anti-tumor, anti-diabetic, anti-viral, and anti-oxidative [31,32]. Allantoin is a heterocyclic organic compound known as a skin-protectant for its skin-conditioning properties [33]. Extract of *Symphyti radix* was used in this research as a precursor for silver nanoparticle (AgNP) biosynthesis. AgNPs have been a center of interest for many researchers because of their promising results in terms of their antimicrobial, antifungal, and anti-inflammatory properties [13,34].

The present paper reports the synthesis of natural polymer films with green silver nanoparticles using aqueous root extracts of *Symphyti radix*, evaluating the composite’s potential applications as an antibacterial material against Gram-negative and Gram-positive pathogenic bacteria strains, with antioxidant potential. 

## 2. Materials and Methods

### 2.1. Chemicals

Silver nitrate (AgNO_3_), ABTS^•+^ (2,2′-azino-bis(3-ethylbenzothiazoline-6-sulphonic acid)), sodium acetate trihydrate (CH_3_COONa × 3H_2_O), iron(III) chloride hexahydrate (FeCl_3_ × 6H_2_O), TPTZ (2,4,6-Tris(2-pyridyl)-*s*-triazine), TFPH (trifluoperazine hydrochloride), and Trolox (6-hydroxy-2,5,7,8-tetramethylchroman-2-carboxylic acid) were purchased from Merck (Darmstadt, Germany); ethanol (96.3% *v*/*v*) was obtained from Stumbras, AB (Kaunas, Lithuania); potassium chloride (KCl) was obtained from Scharlau (Barcelona, Spain); potassium bisulfate (K_2_S_2_O_8_) and DPPH^•^ (2,2-Diphenyl-1-(2,4,6-trinitrophenyl)hydrazin-1-yl) were obtained from Alfa Aesar GmbH & Co KG (Karlsruhe, Germany); sulfuric acid (H_2_SO_4_) 95% was purchased from Chempur (Piekary Śląskie, Poland). All chemicals used were of analytical grade.

### 2.2. Plant Materials

Finely cut *Symphyti radix* (Švenčionių vaistažolės UAB, Švenčionys, Lithuania) was purchased from a pharmacy of the Lithuanian University of Health Science operating in Sukilėlių street. 13, Kaunas (Lithuania). Using a mill, plant roots were ground to a powder (IKA^®^ A11 basic, Staufen, Germany). Loss on drying before analysis was determined by drying about 1 g of powdered roots in a moisture analyzer (Precisa HA 300, Precisa Instruments AG, Dietikon, Switzerland) until complete evaporation of water and volatile compounds at a drying temperature of 105 °C. The data were recalculated for absolute dry weight (DW).

### 2.3. Preparation of Roots Extracts

Fifty grams of dried *Symphyti radix* powder were poured into 200 mL of distilled water and left to swell for 3 h at room temperature. Further on, the resulting mixture was stirred in a magnetic stirrer at a temperature of +55 °C for 2 h. The resulting natural polymer matrix was strained, and the extract was further centrifuged. A clear, even, slightly yellowish extract with a gel consistency was obtained.

### 2.4. Green Synthesis of Silver Nanoparticles and Polymer Film Formatting

Different amounts of AgNO_3_ were dissolved: 1 mg, 2 mg, 3 mg, and 4 mg in 5 mL of distilled water, and were mixed with 50 mL of *Symphyti radix* aqueous extracts under vigorous stirring at +50 °C for 3 h. The mixtures were incubated in the dark at room temperature for 24 h. After this, the obtained AgNP colloids were cast onto a polyethylene film. The obtained natural films with AgNPs were dried for 18 h at a temperature of 65 °C to form a film. Obtained samples of pure film *Symphyti radix* (*Sym. Radix*); *Sym. Radix*/AgNPs1 (1 mg in matrix AgNO_3_); *Sym. Radix*/AgNPs2 (2 mg in matrix AgNO_3_); *Sym. Radix*/AgNPs3 (3 mg in matrix AgNO_3_); and *Sym. Radix*/AgNPs4 (4 mg in matrix AgNO_3_).

### 2.5. Determination of Total Phenolic Content

The total phenolic content (TPC) of the tested samples was determined by Folin–Ciocalteu’s method [18], using gallic acid as the standard. The spectrophotometer used was a Cintra 202 (GBC Scientific Equipment, Melbourne, Australia) and the absorbance was measured at 765 nm for the samples. The total phenolic content was determined using a gallic acid calibration curve and was expressed as mg of gallic acid equivalent (GAE) per 100 g dry weight (mg GAE/100 g DW).

### 2.6. Determination of Antioxidant Activity

The scavenging activity of ABTS^•+^ was established as reported by the method of Re [35], with some modifications. A volume of 2 mL of ABTS^•+^ solution (absorbance 0.800 ± 0.02) was mixed with 20 μL of the tested samples. The absorbance of each sample was measured at 734 nm using a Cintra 202 spectrophotometer (GBC Scientific Equipment, Melbourne, Australia) after 30 min.

The determination of DPPH^•^ free radical scavenging activity was conducted in accordance with the method suggested by Brand Williams, Cuvelie, and Berset [36], with some modifications [37]; 2 mL DPPH^•^ solution in 99.0% *v*/*v* ethanol was mixed with 20 μL of the tested samples. A decrease in absorbance at 515 nm was measured using a Cintra 202 spectrophotometer (GBC Scientific Equipment, Melbourne, Australia) after 30 min.

The ferric-reducing antioxidant power (FRAP) assay was determined as depicted by Benzie and Strain [38], with some modifications. The FRAP solution was prepared by mixing TPTZ (0.01 M in 0.04 M HCl), FeCl_3_ × 6H_2_O (0.02 M in water), and acetate buffer (0.3 M, pH 3.6) at a ratio of 1:1:10. A volume of 2 mL of a recently prepared FRAP reagent was mixed with 2 μL of sample. The absorbance increase was measured at 593 nm using a Cintra 202 spectrophotometer (GBC Scientific Equipment, Melbourne, Australia) after 30 min.

All antioxidant activity assays were carried out using Trolox calibration curves and were expressed as μmol of the Trolox equivalent (TE) per one gram of dry weight (µmol TE/g DW).

### 2.7. Scanning Electron Microscopy (SEM) and Transmission Electron Microscopy (TEM) Analysis

Scanning electron microscopy (SEM–EDS). The surface morphology and chemical analysis of the investigated composites with AgNPs were investigated by SEM “FEi Quanta 200 FEG”: resolution—1.2 nm, accelerating voltage—20 kV (FEI Company, Hillsboro, OR, USA). The EDS system consists of a Bruker XFlash^®^ 4030 (FEI, Hillsboro, OR, USA) X-ray energy dispersive detector, signal processor, controller, and ESPRT 2.1 data analysis software. The spectrometer allows for the quantitative and qualitative assessment of the chemical composition of the sample by detecting chemical elements in the area of the sample and determining the distribution map of individual chemical elements on the surface. Additionally, this study allows for the qualitative assessment of the morphology and structural changes of the object under study. Samples were scanned at least three different times. 

Transmission electron microscopy (TEM). TEM “Tecnai G2 F20 X-TWIN” (FEI, Hillsboro, OR, USA) studied the size, distribution, and structure of nanoparticles. A Schottky-type field emission electron source was used, and an accelerating voltage of 20–200 kV was applied. The resolution of the microscope was 0.8–1.0 nm. An EDAX Spectrometer with r-TEM detector and 11 MPix ORIUS SC1000B (Gatan Inc., Pleaston, CA, USA) CCD camera were used. Point/line resolution—0.25/0.102 nm.

### 2.8. Physicochemical Characterization

The formation of *Sym. Radix*/AgNPs 1 was checked using a Lambda 25 UV-vis spectrometer (PerkinElmer, Waltham, MA, USA). The analysis was performed in a wavelength range of 200 to 800 nm. Ultrapure water was used as the blank for the UV-Vis experiments.

### 2.9. Antimicrobial Activity

The antimicrobial activity of the *Symphyti Radix* pure biofilm and the composites with the synthesized silver nanoparticles were investigated against Gram-negative and Gram-positive bacteria cultures. The agar diffusion was chosen for the evaluation of antibacterial activity. The antimicrobial activity of extracts was tested via an agar well diffusion assay. For this purpose, a 0.5 McFarland Unit density suspension (~10^8^ CFU mL^1^) of each pathogenic bacterial strain was inoculated onto the surface of cooled Mueller Hinton Agar (Oxoid, UK) using sterile cotton swabs. Wells of 6 mm in diameter were punched in the agar and filled with sample. The experiments were repeated three times, and the average size of the inhibition zones was calculated. Antimicrobial activity against the tested bacteria was determined by measuring the diameter of the inhibition zones (mm). The antimicrobial activity of composites was determined against *Staphylococcus aureus* (*S. aureus*) ATCC 25923, *Beta hemolytic streptococcus group b* (*ß-streptococcus*) ATCC 15185, *Staphylococcus epidermidis* (*S. epidermidis*) ATCC 12228, *Enterococcus faecalis* (*E. faecalis*) ATCC 29212, *Escherichia coli* (*E. coli*) ATCC 25922, *Klebsiella pneumoniae* (*K*. *pneumoniae*) ATCC 13883, *Pseudomonas aeruginosa* (*P. aeruginosa*) ATCC 27853, *Proteus vulgaris* (*P. vulgaris*) ATCC 8427, *Bacillus cereus* (*B. cereus*) ATCC 11778, and *Candida albicans* ATCC 10231 in the Lithuanian University of Health Sciences (Kaunas, Lithuania).

### 2.10. Determining the Strength of the Film

The film strength was determined with a TA.XTPlus texture analyzer (Stable Micro Systems, London, UK) using a P/2 2 mm diameter probe. Analytical conditions: the measurement started when the probe was in contact with the surface of the sample, and a force of 1 g was recorded; the probe struck the sample at a speed of 1 mm/s until it pierced it. Three films of each variant were taken for analysis. The analytical data were processed using Texture Exponent software (Version 6.0, Stable Micro Systems, London, UK).

### 2.11. Statistical Analysis

The experiments were carried out in triplicate, and all the results were expressed as mean value ± standard deviation (SD). To identify significant differences (*p* < 0.05), one-way ANOVAs followed by Turkey’s HSD test was performed using the statistical package GraphPad Prism 8 software (GraphPad, San Diego, CA, USA).

## 3. Results and Discussion

### 3.1. Determination of Total Phenolic Content and Antioxidant Activity

As part of our research, we evaluated the total phenolic content (TPC) and three distinct in vitro radical scavenging activities (ABTS, DPPH, and FRAP) of extracts derived from the *Symphyti radix*. Plant extracts are rich in biologically active compounds that exhibit unique antioxidant mechanisms. However, due to the complex nature of these natural matrices, a single methodology may not be sufficient for analyzing their antioxidant capacity [39]. It is highly recommended to use multiple methods to obtain a comprehensive assessment of the antioxidant potential of plant extracts [40,41]. The role of functional groups, such as OH and COOH, in promoting the creation of AgNPs from AgNO_3_ during nanoparticle production is crucial [42,43]. These groups act as both reducing and stabilizing agents. When plant extracts mediate the formation of AgNPs, they can inherit their biological functions. This provides a potential solution for combatting pathogenic bacteria resistant to multiple drugs [44,45].

**Figure 1:**
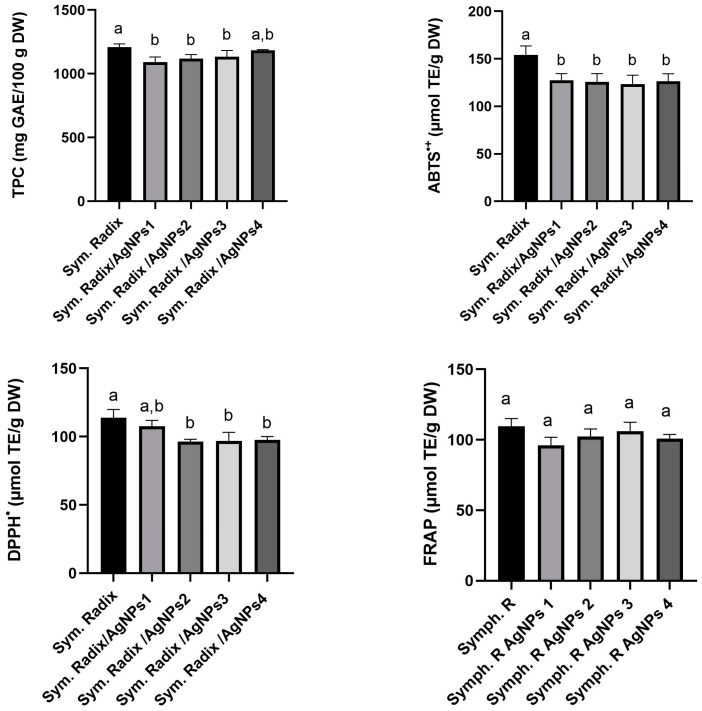
Total phenolic content and in vitro antioxidant capacity using ABTS, DPPH, and FRAP assays of *Sym. Radix* and *Sym. Radix/AgNPs* extracts. Values were expressed as mean ± standard deviation (*n* = 3); different letters indicate statistically significant differences between plant extracts (one-way ANOVA and Tukey’s HSD test, *p* < 0.05).

This experiment aimed to study the antioxidant activity of crude plant extracts obtained from *Symphyti radix* and AgNPs synthesized using these extracts as reducing agents (Figure 1).

The results in Figure 1 show that the TPC content of extracts from *Sym. Radix* and *Sym. Radix*/*AgNPs* reached 1208.6 and 1092.1 mg GAE/100 g, respectively. The observed values of the *Sym. Radix* and *Sym. Radix*/*AgNPs* extracts were slightly lower than Triwan et al.’s [46] indicated TPC value of water extract of 1500.38 mg GAE/100 g, but higher than Sowa et al.’s [27] indicated value of water extract of 600.14 mg GAE/100 g. These differences in TPC values in plants can be attributed to a variety of environmental and non-living factors, such as seasonal changes, water levels, temperature, growth conditions, and light. In addition, the plant’s species and variety also significantly impact the variety and composition of the phytochemicals found in it [47].

The antioxidant capacity of *Sym. Radix* and *Sym. Radix*/*AgNPs* extracts were measured using ABTS^•+^ and DPPH^•^ methods and ranged from 123 to 154 µmol TE/g DW and 93 to 113 µmol TE/g DW, respectively (Figure 1). The FRAP antioxidant activity of the extracts ranged from 96 to 109 µmol TE/g DW. All samples showed significant differences, except for the FRAP radical scavenging activity. The total phenolic content and antioxidant activity were slightly higher in aqueous extracts of *Sym. Radix*.

### 3.2. Structural Analysis of Symphytum officinale Film Silver Nanopartiles

**Figure 2:**
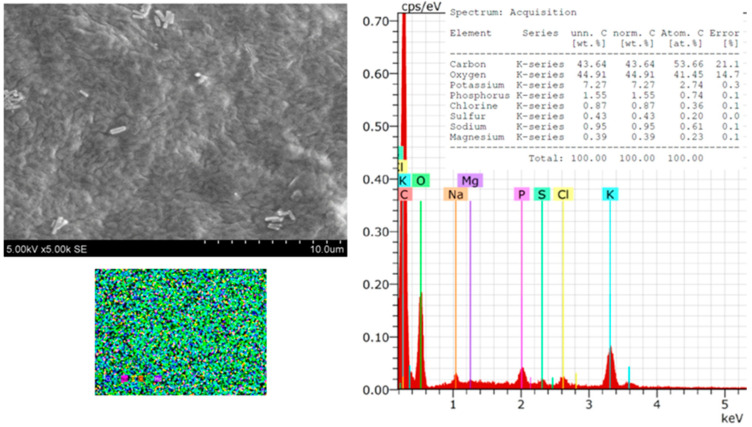
SEM images and EDS spectra and element mapping of pure *Sym. Radix* film.

The morphology, size, shape, and chemical composition of the biosynthesized Symphytum radix pure film and the film with green AgNPs were examined using SEM–EDS. Figure 2 presents SEM microphotographs of the pure Symphytum radix film. The morphological properties of the obtained film can be seen, which is quite even, without cracks or holes. The conducted SEM–EDS studies reveal the chemical composition of the film, which included carbon—43.64, oxygen—44.91, potassium—7.27, phosphorus—1.55, chlorine—0.87, sulfur—0.43, sodium—0.95, and magnesium—0.39 wt.%. All these elements were evenly distributed over the entire tested surface without forming any agglomerates or other aggregates. After the synthesis of the AgNPs, the surface of the film changed. The surface became significantly more even and more uniform; morphological reliefs of uneven thickness were not visible.

**Figure 3:**
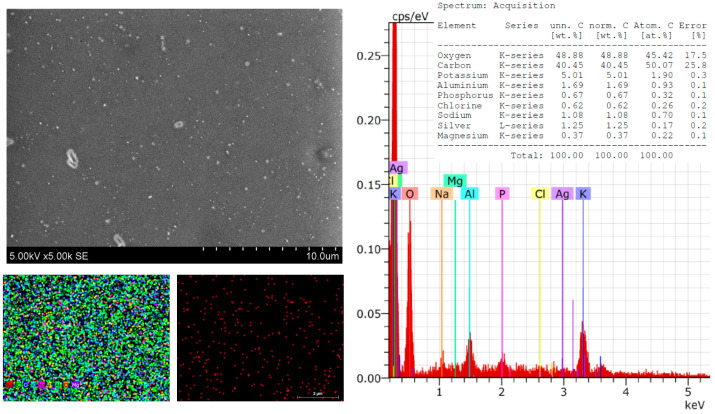
SEM images and EDS spectra and element mapping of *Sym. Radix*/AgNPs1 films.

However, spherical particles, which are unevenly distributed on the surface of the Sym, were clearly visible. *Radix* films were clearly visible (Figure 3). These particles could be associated with the distribution of the AgNP particles, which was confirmed by the presented mapping of the AgNPs. The conducted SEM–EDS studies revealed the chemical composition of the film with the AgNPs, which included carbon—40.45, decreased by 3.19; oxygen—48.88; potassium—5.01, decreased by 2.26; phosphorus—0.67, decreased by 0.88; chlorine—0.62, decreased 0.25; silver—1.25; and magnesium—0.37, decreased 0.02% by mass. These material reductions confirm that they were used during synthesis (Figure 3). The EDS spectra showed peaks at 3.0 keV, which could be attributed to the binding energy of silver and could confirm the formation of AgNPs [1,48].

**Figure 4:**
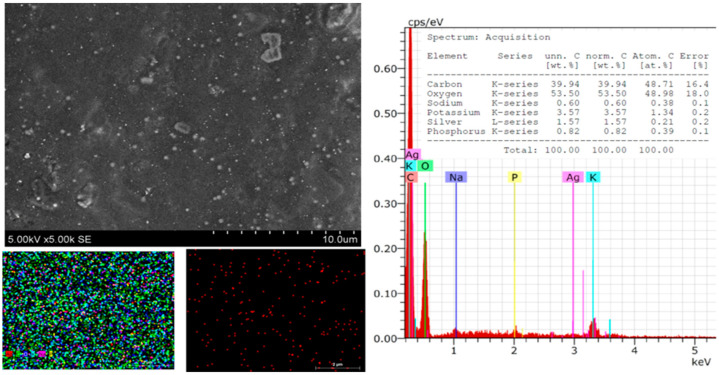
SEM images and EDS spectra and element mapping of *Sym. Radix*/AgNPs2 films.

Significant differences in the morphological surface were not observed after doubling the amount of silver in the composite (Figure 4). Individual particles on the surface that did not tend to form agglomerates were also visible. These could also be associated with green AgNPs. Only a small number of particles were observed, which could be explained by the fact that some AgNPs were in the biocomposite matrix in deeper layers. As in the previously discussed cases, the elemental composition was changed after synthesis.

**Figure 5:**
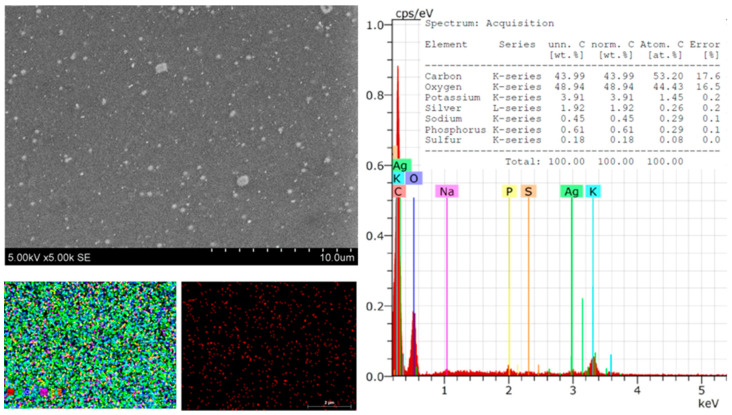
SEM images and EDS spectra and element mapping of *Sym. Radix*/AgNPs3 films.

Element composition: carbon—43.99; oxygen decreased to 4.03; potassium—3.91 (on pure film was 7.27); phosphorus—0.61, decreased by 0.95; sulfur—0.18 (on pure film was 0.43), decrease by 0.25 wt.%; Sodium—0.45, also decreased by 0.5 wt.% (Figure 5). These observations confirm the formation of AgNPs, which increased proportionally with the amount of silver nitrate.

**Figure 6:**
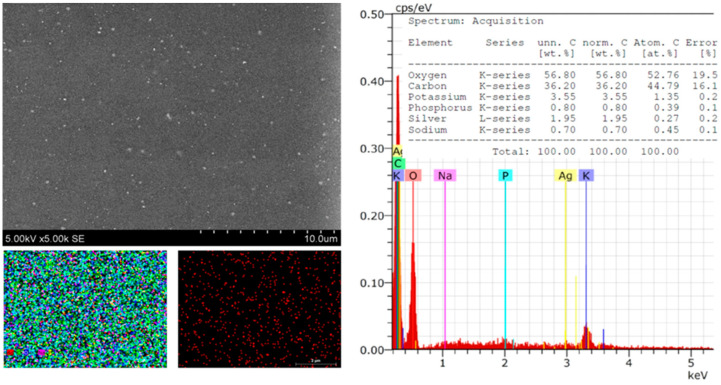
SEM images and EDS spectra and element mapping of *Sym. Radix*/AgNPs4 films.

Further on, by increasing the amount of Ag in the composite, it can be seen that the surface morphology had no changes. After the green synthesis, the composite’s elemental composition changed significantly, confirming that the reaction took place. Elemental composition: carbon—36.2 ↓7.44, potassium—3.55 ↓3.72, phosphorus—0.8 ↓0.75, sodium—0.7 ↓0.25, silver 1.95, and magnesium—0 ↓0.39 wt.% (Figure 6).

**Figure 7:**
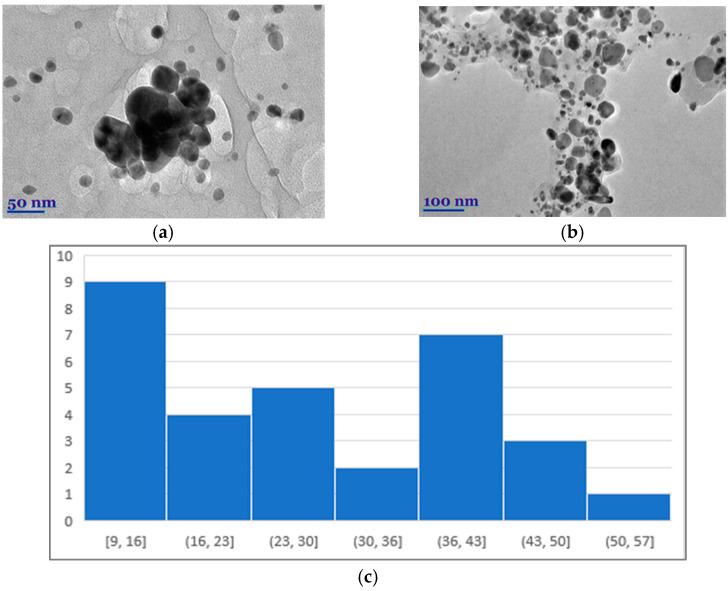
TEM images of biosynthesized *Sym. Radix***/**AgNPs-3 (**a**,**b**) in different magnifications and histogram (**c**).

The size, size distribution, and shape of the metal nanoparticles obtained by green methods were studied by SEM and TEM (Figure 7a,b). It can be seen that the predominant size of the silver nanoparticles was 25.72 nm. However, there were also larger ones, the size of which reached as much as 59 nm (Figure 7).

From the photos presented, it can be seen that the silver nanoparticles had an irregular geometric shape, but were most commonly observed in a spherical shape. A spherical shape is obtained in the case of AgNP particle frequencies obtained by green methods [23,49].

### 3.3. UV-Vis Spectroscopy

**Figure 8:**
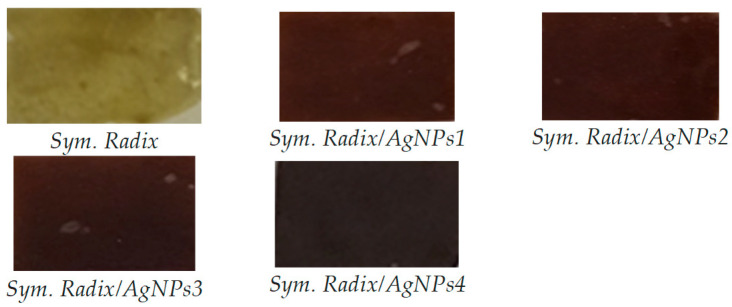
Changes in the color of *Sym. Radix* pure films and with AgNPs.

**Figure 9:**
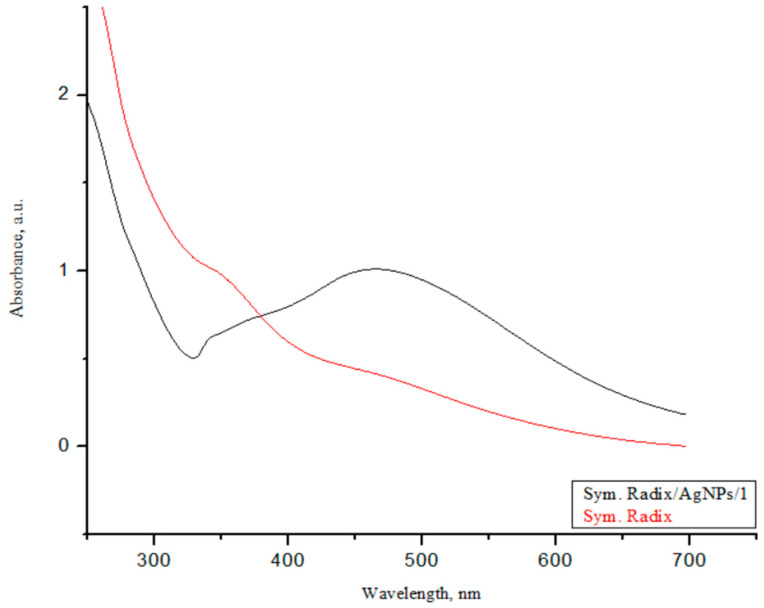
UV-vis absorption spectra of plant extracts and biosynthesized AgNPs.

The successful synthesis of AgNPs using the investigated *Sym. Radix* extracts was confirmed by color changes, and the color change of the colloidal solutions from yellowish to dark brown evidenced the formation of *Sym. Radix*/AgNPs (Figure 8). The color change of the nanoparticle colloidal solutions happened due to localized surface plasmon resonance (LSPR) after the bioreduction of Ag^+^ ions to Ag^0^ by phytochemicals; this was confirmed by UV-visible spectroscopic analysis, as presented in Figure 9.

The biosynthesized *Sym. Radix* that was stabilized with *Sym. Radix* aqua extract showed a strong LSPR peak at 459 nm. Generally, the intensity and position of the LSPR are dependent on the size and shape of nanoparticles and the composition of the surrounding medium [50]. It is proposed that smaller nanoparticles primarily absorb light and have peaks near 400 nm. In contrast, larger spherical particles exhibit increased scattering and have peaks that broaden and shift towards longer wavelengths [51].

### 3.4. Antibacterial Activity

The antibacterial activity of the pure *Sym. Radix and Sym. Radix*/*AgNPs* composites were investigated against both Gram-negative and Gram-positive bacterial strains. The results are presented in Table 1. It can be seen that all samples with AgNPs exhibited antimicrobial activity against all tested bacterial strains. The pure *Sym. Radix*. film showed antimicrobial activity only against strains of *S. aureus* and *ß-streptococcus*, Gram-positive bacteria. This activity can be explained by the rich composition of biologically active compounds in the obtained film [1,3,6].

After synthesizing even a small amount of AgNPs, it can be seen that antimicrobial activity was obtained for all tested Gram-positive and Gram-negative bacterial cultures. The strongest activity was obtained against *ß-streptococcus*, where the zone of inhibition reached 2.55 ± 0.05 mm. In the case of other bacteria, the zones of inhibition varied from 0.5 to 1.80 mm. After further doubling the number of AgNPs in the composite, an apparent increase in antimicrobial activity could be seen. The zones of inhibition increased to ±4 mm for Gram-positive bacteria. A slightly lower microbiological activity was obtained in Gram-negative bacterial cultures, which varied from 1.10 mm, which can be explained by the morphological differences in the bacteria [52].

Even small AgNPs exhibited antimicrobial activity against both Gram-positive and Gram-negative bacterial strains. Numerous scientific studies have shown that silver nanoparticles, in both colloidal and ionic forms, have a wider spectrum of antibacterial effects than most other nanoparticles. The antimicrobial activity of silver ions is obtained by reacting with the main parts of the bacterial cell: the DNA, plasma membrane, cell wall, and proteins [2]. Due to their small dimensions and very high specific surface area, silver nanoparticles adhere firmly to the surface of bacteria. Silver ions, interacting with the bacterial cell membrane and the sulfur compounds in its proteins, damage its functionality and integrity. When damage occurs, AgNPs easily penetrate inside the cell, thus causing DNA damage and disrupting the DNA replication process, which inhibits bacterial proliferation [53].

### 3.5. Strength of the Film

The puncture force was studied in order to find out the influence of silver nanoparticles on the mechanical behavior of the films. From the data presented in Table 2, it can be seen that the presence of silver nanoparticles significantly increased the puncture force.

In the first case, when the film without metal nanoparticles was tested, the force value was 45.9 puncture force, g, but after introducing even a small number of AgNPs, this force increased by up to five times. After further doubling the number of AgNPs, this force increased evenly and increased by even ~9.7 times compared to the film without AgNPs. However, as the amount of silver continued to increase, this force began to decrease; however, it still remained up to three times higher compared to the film without AgNPs. This can be explained by the fact that, after introducing even small amounts of Ag, the film structure becomes elastic, but when higher concentrations are reached, this force tends to decrease due to morphological changes in the film [48,49,54,55].

## 4. Conclusions

Functional natural polymer films with green AgNPs were synthesized using a sustainable, environmentally friendly, and economical method using plant root extracts of *Symphyti radix* as a reducing and capping agent. The results suggest that the root extracts affected the morphology of the nanoparticles. The size ranges of AgNPs mediated by *Symphyti radix* extracts were found to be 17.5 nm and 34.3 nm, respectively. Antioxidant activity and total phenolic content were slightly higher in aqueous extracts of *Sym. Radix*. Phenolic compounds found in root extracts and capped AgNPs were responsible for this biological activity and antioxidant activity. Furthermore, the obtained functional polymer films with silver nanoparticles exhibited pronounced antioxidant and broad antimicrobial effects on all tested Gram-positive and Gram-negative bacterial strains and could be promising materials with potent antimicrobial activity.

## Figures and Tables

**Table 1:** The antibacterial activity of pure *Sym. Radix* and *Sym. Radix*/*AgNP* films.

Reference Cultures of Microorganisms	*Sym.* *Radix*.	*Sym. Radix*/	*Sym. Radix*/	*Sym. Radix*/	*Sym. Radix*/
		AgNPs1	AgNPs2	AgNPs3	AgNPs4
	Inhibition zone (mm)				
*S. aureus*	1.50 ± 0.05 ^e^	1.80 ± 0.01 ^d^	3.40 ± 0.10 ^c^	5.05 ± 0.05 ^b^	5.20 ± 0.05 ^a^
*ß-streptococcus*	1.40 ± 0.10 ^e^	2.55 ± 0.05 ^d^	4.12 ± 0.25 ^c^	5.20 ± 0.00 ^b^	5.95 ± 0.01 ^a^
*S. epidermidis*	0.00 ± 0.00 ^e^	1.00 ± 0.10 ^d^	1.80 ± 0.30 ^c^	4.05 ± 0.00 ^b^	5.40 ± 0.10 ^a^
*E. coli*	0.00 ± 0.00 ^e^	1.00 ± 0.40 ^d^	1.65 ± 0.01 ^c^	2.95 ± 0.50 ^b^	4.10 ± 0.20 ^a^
*K. pneumoniae*	0.00 ± 0.00 ^d^	0.50 ± 0.25 ^c^	1.10 ± 0.50 ^c^	2.50 ± 0.20 ^b^	3.10 ± 0.05 ^a^
*P. aeruginosa*	0.00 ± 0.00 ^e^	1.00 ± 0.01 ^d^	2.10 ± 0.01 ^c^	3.80 ± 0.15 ^b^	4.60 ± 0.15 ^a^
*P. vulgaris*	0.00 ± 0.00 ^e^	1.20 ± 0.05 ^d^	2.05 ± 0.65 ^c^	4.50 ± 0.01 ^b^	5.35 ± 0.25 ^a^
*B. cereus*	0.00 ± 0.00 ^e^	1.30 ± 0.00 ^d^	1.95 ± 0.15 ^c^	3.45 ± 0.10 ^b^	4.70 ± 0.01 ^a^
*E. faecalis*	0.00 ± 0.00 ^e^	0.50 ± 0.01 ^d^	1.15 ± 0.01 ^c^	2.85 ± 0.05 ^b^	4.05 ± 0.05 ^a^
*C. albicans*	0.50 ± 0.10 ^c^	0.75 ± 0.25 ^c^	1.05 ± 0.01 ^b^	3.10 ± 0.50 ^a^	3.70 ± 0.10 ^a^

Different superscript letters in the same line indicate statistically significant differences between Gram-positive and Gram-negative bacteria strains of plant extracts (*p* < 0.05).

**Table 2:** Strength of the film *Sym. Radix* and *Sym. Radix*/*AgNP* composites.

Sample	Puncture Force, g
*Sym. Radix*.	45.9 ± 1.67 ^e^
*Sym. Radix*/*AgNPs1*	236.0 ± 9.13 ^b^
*Sym. Radix*/*AgNPs*2	449.8 ± 23.26 ^a^
*Sym. Radix*/*AgNPs*3	125.0 ± 5.10 ^d^
*Sym. Radix*/*AgNPs4*	154.4 ± 4.01 ^c^

Different superscript letters in the same line indicate statistically significant differences between the antioxidant activity of plant extracts with AgNPs in films (*p* < 0.05).

## Data Availability

All data generated during this study are included in this article.
